# The ER Is a Common Mediator for the Behavior and Interactions of Other Organelles

**DOI:** 10.3389/fpls.2022.846970

**Published:** 2022-03-25

**Authors:** Jaideep Mathur, Olivia Friesen Kroeker, Mariann Lobbezoo, Neeta Mathur

**Affiliations:** Laboratory of Plant Development and Interaction, Department of Molecular and Cellular Biology, University of Guelph, Guelph, ON, Canada

**Keywords:** ER, organelle extensions, fluorescent proteins (FPs), chloroplasts, peroxisomes, mitochondria

## Abstract

Optimal functioning of a plant cell depends upon the efficient exchange of genetic information, ions, proteins and metabolites between the different organelles. Intuitively, increased proximity between organelles would be expected to play an important role in facilitating exchanges between them. However, it remains to be seen whether under normal, relatively non-stressed conditions organelles maintain close proximity at all. Moreover, does interactivity involve direct and frequent physical contact between the different organelles? Further, many organelles transition between spherical and tubular forms or sporadically produce thin tubular extensions, but it remains unclear whether changes in organelle morphology play a role in increasing their interactivity. Here, using targeted multicolored fluorescent fusion proteins, we report observations on the spatiotemporal relationship between plastids, mitochondria, peroxisomes and the endoplasmic reticulum in living plant cells. Under normal conditions of growth, we observe that the smaller organelles do not establish direct, physical contacts with each other but, irrespective of their individual form they all maintain intimate connectivity with the ER. Proximity between organelles does increase in response to stress through concomitant alterations in ER dynamics. Significantly, even under increased proximity the ER still remains sandwiched between the different organelles. Our observations provide strong live-imaging-based evidence for the ER acting as a common mediator in interactions between other organelles.

## Introduction

The eukaryotic cell is characterized by the presence of functionally discrete domains called organelles. The mitochondria and chloroplasts are distinguished by the presence of an envelope and postulated to be of endo-symbiogenic origins (Mereschkowsky, 1905; Sagan, 1967; Margulis et al., 2006; Martin et al., 2015). By contrast, organelles such as peroxisomes, Golgi bodies, lipid droplets, vacuoles, and assorted vesicles are *de novo* derivatives of the endomembrane system and the bounding plasma-membrane. While each organelle creates a biochemically independent domain within the cell, it is clear that the optimal functioning of the cell as a unit relies upon the combined activities of all constituent organelles. Organelle interactions provide succinct explanations for the extensive biochemical and genetic exchanges known to occur between organelles. However, from a cell biological perspective it is not always clear whether the interactions involve direct physical contacts between the different organelles or are only possible in an indirect manner through mediation of vesicles, protein complexes and acyl-lipids. In recent years it has become increasingly clear that discrete membrane contact sites (MCS) between organelles facilitate inter-organelle trafficking (Prinz, 2014; Michaud et al., 2016; Michaud and Jouhet, 2019). Notably, MCS form between closely appressed membranes, typically less than 30 nm apart (Helle et al., 2013; Prinz, 2014). Thus, a key requirement for membrane interactions to occur is for two organelles to be very close to each other. In plants, organelle proximity leading to direct interactivity has been best documented for the fusion of independent mitochondria (Schattat et al., 2012) and is considered common for membrane vesicles fusing together or fusing with other organelle membranes (reviewed by Perico and Sparkes, 2018; Oikawa et al., 2019). Similarly, membrane fusion and detachment form the underlying basis for the dynamic behavior of the endoplasmic reticulum (ER; Staehelin, 1997; Stefano et al., 2014). It is worth noting that while molecular exchanges between endomembrane derivatives may generally involve two membrane bilayers, for endosymbiont organelles the presence of a two-layered envelope necessitates the crossing of four bi-layered membranes. Although the presence of ion channels and gated pores, specific transporters and dedicated translocons facilitates inter-organelle exchanges physical proximity between organelles is believed to play an important role in their interactions (Helle et al., 2013; Mehrshahi et al., 2013, 2014).

However, a critical assessment of published literature suggests that in many cases the so called “interactions” between organelles are not built on actual data involving physical connectivity between organelles. Instead, they are inferences drawn from correlated biochemical findings that rely on clean preparations obtained by efficiently separating the different organelles from the cellular milieu. Consequently, actual physical contacts between organelle pairs such as chloroplasts and mitochondria, mitochondria and peroxisomes, and chloroplasts and peroxisomes have not been unequivocally demonstrated.

Nevertheless, the close proximity of chloroplasts, mitochondria and peroxisomes, three biochemically linked organelles (Bauwe et al., 2010), is succinctly depicted through several classic transmission electron microscopic images such as those presented in Frederick and Newcomb (1969) and Frederick et al. (1975). These images depict mitochondria and cytosomes (later identified as peroxisomes) appressed to chloroplasts and are annotated by definitive statements such as “mitochondria are abundant around the chloroplasts. The cytosomes are similarly distributed but show a greater tendency to lie wedged tightly between adjacent chloroplasts” (Frederick and Newcomb, 1969). While noting a lack of ER membranes between the three organelles, the authors cautioned that the images of mesophyll cells in photo-respiring plants might not be representative for other parts of plants and other plant species (Frederick and Newcomb, 1969). Several other TEM based studies have pointed to membrane continuities between chloroplasts and different organelles (Gibbs, 1962; Diers, 1966; Cran and Dyer, 1973; Crotty and Ledbetter, 1973). In more recent times comparative observations on the proximity between chloroplasts, peroxisomes, and mitochondria in wild type Arabidopsis and photorespiration defective mutants of the Arabidopsis PEROXIN10 (PEX10) gene (Schumann et al., 2007) have further strengthened the idea of organelle juxtaposition as a major factor in facilitating organelle interactions. Similarly, the implications of organelle proximity have been used for insights on the functional anatomy of rice leaves (Sage and Sage, 2009). TEM based interpretations of closely appressed membranes have also been made for glyoxysomes and lipid droplets (Hayashi et al., 2001). Notably, all TEM images are obtained from fixed, dead plant tissue and therefore do not portray the dynamic nature of living plant cells. Whether organelle proximity is a common occurrence in living plant cells under normal conditions of growth and development has thus remained unclear.

Advances in the imaging of living cells and analyzing subcellular dynamics using video rate -cinephotomicrography (Wildman et al., 1962; Gunning, 2005) and time-lapse movies of fluorescently highlighted organelles have shown the continuous movement of different organelles in living plant cells. However, in this dynamic subcellular milieu it is unclear as to how close two organelles should be, and for how long they should remain together, for an interaction to occur. Upon observing living plant cells, our attention is readily drawn to organelle clusters and to sporadic occurrences of two or more organelles remaining near each other over relatively long periods even though the majority of organelles around the clusters may continue moving independently. Studies that take the majority behavior into account and provide relevant comparative data are largely missing. Moreover, light microscopy-based imaging does not provide the ultrastructural resolution afforded by TEM images. The limits on image resolution become very relevant in fluorescence-based microscopy since the blooming effect of fluorescent probes can often create a false impression of organelle proximity and not allow an estimation of the true distance between them. Nevertheless, imaging of living cells has provided strong correlations between organelle motility, cytoskeletal elements, and motor proteins (Nebenführ et al., 1999; Logan and Leaver, 2000; Brandizzi et al., 2002; Mano et al., 2002; Mathur et al., 2002; Sparkes et al., 2011; Stefano et al., 2014; Oikawa et al., 2015). In addition, it has become clear that organelle morphology and behavior in plant cells are not fixed traits and can change transiently during rapid organelle responses to environmental cues (Mathur, 2020). The sporadic changes in organelle shape may involve the formation of thin, tubular, dynamic extensions named stromules, matrixules, and peroxules from plastids, mitochondria, and peroxisomes, respectively (Köhler and Hanson, 2000; Scott et al., 2007; Sinclair et al., 2009; Mathur, 2021). Plastids are known to aggregate around the nucleus in expanding as well as wounded plant cells, and observations on stromules extended from the plastids aggregated around nuclei have been used to suggest their interactions (Kwok and Hanson, 2004a; Caplan et al., 2015). Proximity-based conclusions have been used to suggest that stromules interact with mitochondria (Kwok and Hanson, 2004b) and other organelles (Natesan et al., 2009; Sage and Sage, 2009). In a similar manner, under high light stress peroxisomal extensions have been shown to interact with mitochondria (Jaipargas et al., 2016). Many of the spatiotemporal correlations have been drawn from cells that are transiently expressing a particular construct or placed under some kind of stress. The most common, unintended stress is the use of excised leaf sections that can quickly bias observations through wound induced artifacts. Whether similar correlations can be made in unstressed cells remain unclear. In recent years the presence of organelle extensions and tubular organelles has become well established and clearly extends the interactive surface of an organelle beyond the diameter of the main body (Mathur, 2021). Whether plastid extensions interact with matrixules or peroxules, or if the tubular forms of organelles interact more with each other than when they are present in a spheroidal form, remains unclear.

Here we have investigated whether it is common for chloroplasts, mitochondria, and peroxisomes to come in direct physical contact with each other in living plant cells under normal conditions of growth and development. Our investigations have utilized several stable transgenic lines of Arabidopsis expressing fluorescent proteins targeted to the different organelles (Table 1). As our investigations progressed, the need for considering another organelle that may act as a mediator between all other organelles was felt, thus fluorescent probes highlighting the ER lumen and membrane were added. Several Arabidopsis mutants with aberrant morphologies and behavior for the different organelles were also utilized in our investigations.

**TABLE 1:** Transgenic Arabidopsis plants and mutants used in the study.

Targeted Organelle(s)	Name used for fluorescent fusion protein/color	Characteristic feature(s) of transgenic line	References
**Single transgenic**			
Plastid (stroma)	tpFNR-EGFP/G	Stroma and stromules green	Schattat et al., 2011a
Plastid (stroma)	Pt-YK/Y	ABRC stock CS16267; pea RUBISCO small subunit fused to EYFP, stroma – stromules yellow	Nelson et al., 2007
Plastid (OEM)	SFR2-mRFP/R	Highlights plastid OEM in red fluorescence	∙ This study
Peroxisome matrix	YPeroxi/Y	Yellow fluorescent peroxisomes and peroxules YFP-SKL	Mathur et al., 2002
Peroxisome matrix	GPeroxi/G	Green fluorescent peroxisomes due to GFP-SKL	Mano et al., 2002
Mitochondrion matrix	mitoGFP/G	Green fluorescent mitochondria and matrixules due to targeting using a tpßATPase- GFP fusion protein	Logan and Leaver, 2000
Mitochondrion matrix	Mt-YK = Ymito/Y	ABRC stock CS16264; mitochondrial targeting sequence from yeast ScCOX4 fused to EYFP	Nelson et al., 2007
Endoplasmic reticulum (ER) lumen	GER/G	Green ER with GFP filled lumen due to ER targeting signal sequence appended to mGFP-HDEL	Haseloff et al., 1997
Endoplasmic reticulum (ER) lumen	RER/R	Red fluorescent ER with mRFP filled lumen by fusion of ss-mRFP-HDEL	Sinclair et al., 2009
**Double and Triple transgenic**			
Plastid stroma + mitochondrion	tpFNR-EGFP – mtyk/G, Y	Plastids appear green, mitochondria yellow	∙ This study
Plastid OEM + mitochondrion	SFR2mRFP – mitoGFP/R, G	Plastid OEM fluoresces red, mitochondria green	∙ This study
Plastid stroma + peroxisome	tpFNR-EGFP – Yperoxi/G/Y	Plastids appear green peroxisomes yellow	Barton et al., 2013
Plastid stroma + ER	tpFNR-EGFP- RER/G, R	Plastids appear green, ER lumen red fluorescent	Schattat et al., 2011a
Plastid OEM + ER	SFR2mRFP-GER/R, G	Plastid OEM red, ER lumen green fluorescent	∙ This study
Plastid OEM + peroxisome + mitochondrion	SFR2mRFP- GPeroxi – Ymito/R, G,Y	Plastid OEM red, peroxisomes yellow, mitochondria green fluorescent	∙ This study
Plastid + peroxisome + ER	tpFNR-EGFP - Yperoxi – RER/G, Y,R	Plastid stroma green, peroxisomes yellow, ER red fluorescent	Barton et al., 2013, 2014
Peroxisomes + ER	YPeroxi-GER/Y, G	Normal peroxisomes yellow, ER green fluorescent	Barton et al., 2013, 2014
Mitochondrion + ER	mitoGFP-RER/G, R	Normal mitochondria green, ER red fluorescent	Jaipargas et al., 2015, 2016
Mitochondrion - peroxisomes	mtYK -GPeroxi	Yellow fluorescent mitochondria and green peroxisomes	Jaipargas et al., 2015, 2016
Peroxisomes – mitochondrion - ER	GPeroxi-YMito-RER/Y, G, R	Normal peroxisomes Green, mitochondria yellow, ER red fluorescent	Jaipargas et al., 2015; Delfosse et al., 2016
Mitochondrion-Peroxisome - ER	Gmito-Yperoxi-RER/G, Y, R	Normal mitochondria Green, peroxisomes yellow, ER red fluorescent	Barton et al., 2013; Jaipargas et al., 2015
**Mutation and transgene**			
*apm1*	*At4g33650; aberrant peroxisome morphology1/G*	Green fluorescent, elongated peroxisomes and mitochondria due to impaired fission	Mano et al., 2004; Arimura et al., 2004
*apm1*-RER	/G, R	Elongated peroxisomes and red ER lumen	Sinclair et al., 2009; Barton et al., 2013, 2014
*apm1*-MtYK	/G, Y	Green elongated peroxisomes, yellow elongated mitochondria	∙ This study
*apm1*-MtYK – SFR2mRFP	/G, Y, R	Elongated Green peroxisomes and yellow mitochondria, red plastid OEM	∙ This study
*apm1*-MtYK – RER	/G, Y, R	Green elongated peroxisomes, yellow elongated mitochondria, red ER lumen	∙ This study
*arc6*	*At5g42480 accumulation and replication of chloroplasts1/*-	Enlarged mesophyll chloroplasts, detected by chlorophyll autofluorescence	Pyke et al., 1994
*arc6*-tpFNR EGFP	/G	Green stroma in large plastids, many stroma-filled extensions	Delfosse et al., 2016
*arc6* tpFNREGFP-RER	/G, R	Green stroma in large plastids and red ER lumen	Mathur, 2021
*arc6* tpFNREGFP-RER – YPeroxi	/G, R, Y	Green stroma in large plastids, red ER lumen and yellow peroxisomes	∙ This study
*elm1*	*At5g22350 elongated mitochondria1/G*	Elongated, green tubular mitochondria	Arimura et al., 2008
*elm1*-RER	/G, R	Tubular mitochondria and red ER lumen	Jaipargas et al., 2015

*EGFP, G-enhanced green fluorescent protein; YFP, Y-enhanced yellow fluorescent protein; ER, endoplasmic reticulum; mRFP, R-monomeric red fluorescent protein; OEM, plastid outer envelope membrane; tp, transit peptide.*

Combining these various resources have resulted in a comprehensive investigation that provides fresh insights on organelle interactivity in the plant cell.

## Results

### Establishing Baseline Imaging Considerations

When considering the proximity between organelles and trying to link it to possible interactivity and exchanges between them, an important consideration was maintained. Both plastids and mitochondria have envelopes consisting of two membrane bilayers separated by an intermembrane region. Thus, any direct interactions between mitochondria and chloroplasts would be expected to involve at least four membrane bilayers. In comparison, the peroxisome is bounded by a single bilayer membrane only. Therefore, peroxisome interactions with the other two organelles would involve three bilayer membranes. Since earlier studies on organelle interactions have not adequately acknowledged the presence of several membrane layers between organelles most suggestions of their direct interactivity become debatable. Given the scarcity of information, the published TEM images depicting proximity between chloroplasts, mitochondria, and peroxisomes (Frederick and Newcomb, 1969; Schumann et al., 2007; Sage and Sage, 2009) became baseline criterion for organelle interactivity even in our live-imaging based investigations. We looked for instances where two or three of the organelles could be observed together. An implicit bias in our observations became apparent quickly as our eyes readily picked out organelle clusters within the dynamic cytoplasmic strands while ignoring the non-clustered organelles.

The bias was minimized by considering “dwell time”, which denoted the time spent by two organelles in close proximity to each other. The dwell time estimations were made by recording the time that two organelles actually spent with each other within a region circumscribed by the length of the long axis of the smaller organelle (more details in “Materials and Methods” Section).

**FIGURE 1:**
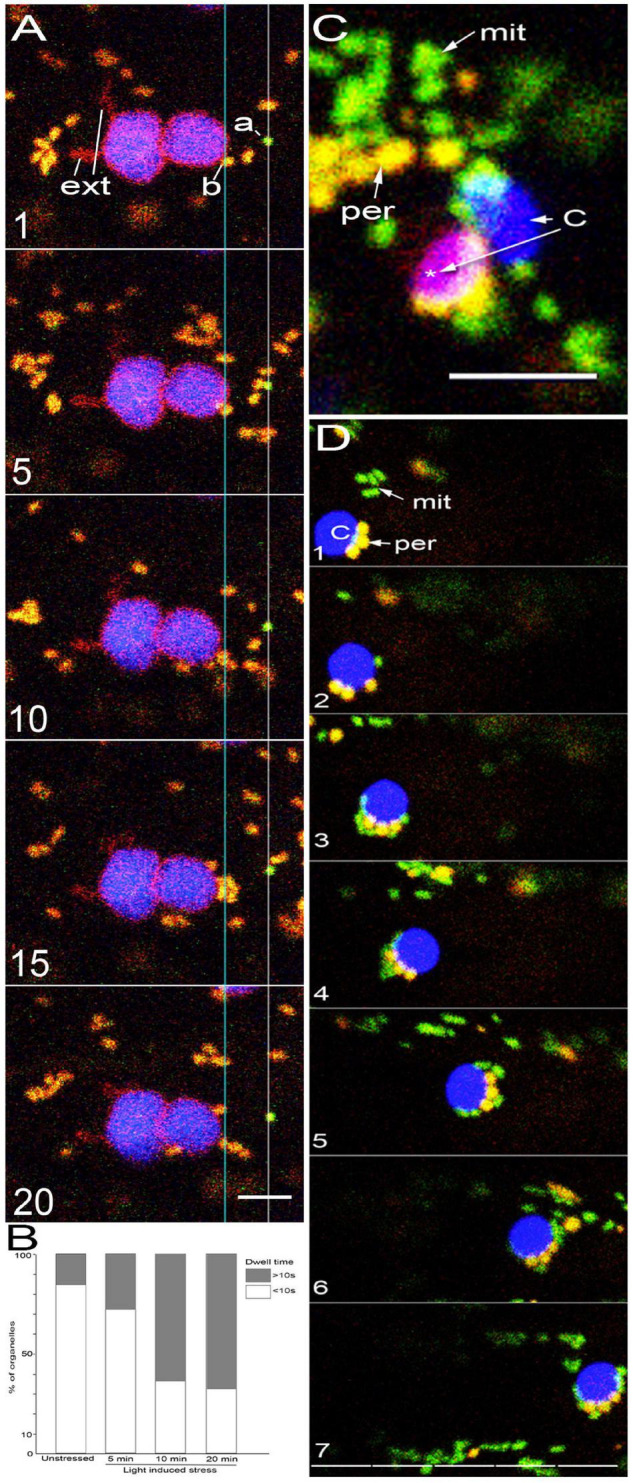
Several different possibilities for organelle interactions are observed in living cells. **(A)** Five snapshots used to illustrate the ideas of coincidental interactions, sustained proximity based on long dwell time and no possible direct interactions when chloroplasts, peroxisomes and mitochondria clearly remain at a distance from each other. The consecutive images are from 100 frames representing about 200 s of imaging (Supplementary Movie 1). A peroxisome labeled “a” maintains its position at a distance from the two chloroplasts observed for 100 frames. A mitochondrion labeled “b” maintains a position at or within 500 nm of the plastid OEM for 100 frames or a total dwell time of 200 s. During this time up to 6 other mitochondria cluster near it but the cluster is maintained only for a maximum of 45 frames or dwell time of 90 s. Other than one mitochondrion that is present close to the plastid OEM during the 200 s, the rest do not exhibit sustained proximity with the plastid or with a peroxisome. The single instance of a peroxisome and mitochondria appearing in frame 15 and the several frames with mitochondrial clusters are considered coincidental occurrences. Notably, two extensions (ext) are present in the chloroplast (C) but neither mitochondria or peroxisomes exhibit sustained proximity with them. **(B)** In unstressed plants, on the basis of 100 peroxisome and mitochondrial clusters with chloroplasts a dwell-time of less than 10 s (ca. 5 frames of our confocal scans) was arrived at for 83 clusters while the rest exhibited dwell time longer than 10 s. High light intensity induced stress by exposing plants for 5 min to light of 450 ± 25 μmol m^–2^s^–2^ resulted in the three organelle clusters with a dwell-time greater than 10 s to 29% Longer exposures of 10 and 20 min resulted the three-organelle cluster with dwell-time longer than 10 s to increase to 64 and 67%, respectively. **(C)** Organelles clustered after a 20-min exposure to high light intensity also contain chloroplasts that based on the loss of chlorophyll autofluorescence appear bleached (pink *) compared to an unbleached chloroplast (blue -c). Plastids with degraded chlorophyll often maintained peroxisome clusters for long dwell time. **(D)** Seven consecutive images illustrating high light induced stress for 10 min that leads to increased proximity between the chloroplasts (blue -C), peroxisomes (per – yellow) and mitochondria (mit – green). A split scale bar with each unit representing 10 μm shows that the clustered organelles remained together as they moved for at least 50 μm. Scale bars – A = 5; C, D = 10 μm.

Using these considerations, we used well-characterized fluorescent protein probes targeted to the plastid stroma, mitochondrial outer envelope membrane and matrix, and the peroxisomal matrix (Table 1). However, stroma-targeted fluorescent probes for plastids do not highlight the outer envelope membrane (OEM), which constitutes the outermost boundary of the plastid and the actual interactor with the rest of the cytoplasm. Therefore, a new probe, proCaMV-35S-SFR2mRFP was created. Stable transgenic plants of SFR2mRFP in the Columbia wild type background showed all plastids with a red fluorescent OEM (Figure 1A). Several double and triple transgenic Arabidopsis plants were developed for observing the spatiotemporal relationship between the three key organelles in living cells (Table 1).

### Chloroplasts, Mitochondria, and Peroxisomes Do Not Maintain Proximity Under Normal Growth Conditions

Experiments reported in this section were aimed at establishing the dynamic behavior of the three organelles and carried out on unwounded, living plant cells of the cotyledon and hypocotyl in 8 to 10-day old soil-grown seedlings of the different transgenic lines (Table 1). Cytoplasmic streaming with organelles moving freely was observed in all the cells. In elongated hypocotyl cells (length 100 ± 30 μm; width 18 ± 8 μm), two distinct organelle behaviors and relationships were observed due to the differences in cytoplasmic flow characteristics. Conspicuous transvacuolar strands contained rapidly flowing and continuously splitting and merging cytoplasmic strands wherein organelles with diameters of 0.5 to 1.5 μm moved at velocities ranging from 1.5 up to 3 μm s^–1^. The positions of organelles within these cytoplasmic strands changed continuously. In double transgenic plants for peroxisomes and mitochondria we observed both organelles moving independently and also in clusters of up to 5 μm diameter. Based on counts from 35 hypocotyl epidermal cells and a total of 350 each of peroxisomes and mitochondria we found that 47% of these organelles moved in tandem. Occasionally both organelles clustered transiently (<10 s) around small epidermal chloroplasts of 2.5 ± 1.2 μm diameter (Barton et al., 2018) which also moved as part of the general cytoplasmic stream. While a case could be made that organelles in a cluster are close to each other and therefore maintain the required proximity for direct interactions, we were unable to uncover a pattern for the formation of the clusters or arrive at a specific number of organelles within each cluster. Moreover, judging from the fluorescent color, many clusters contained organelles of one kind only, and thus could include organelles that might be undergoing division. Alternatively, clusters that contained all three organelles under consideration here were also observed. Since organelles continuously joined a cluster or moved away from it, dwell time measurement was applied to clusters with only two different organelles (Figure 1A and Supplementary Movie 1). On the basis of 100 peroxisome and mitochondrial clusters a dwell time of less than 10 s (ca. 5 frames of our confocal scans) was arrived at for 83 clusters while the rest exhibited dwell time longer than 10 s (Figure 1B). Considering that many organelles did not remain clustered we considered transient proximity-dependent interactivity and possible direct connectivity between organelles in the cytoplasmic strands to be coincidental.

The second pattern of organelle movement observed in hypocotyl cells was different from the rapid cytoplasmic flow and was confined to the shallow cortical cytoplasmic sleeve pressing against the plasma-membrane and cell wall. As also documented in earlier studies on peroxisomes (Barton et al., 2013, 2014), mitochondria (Jaipargas et al., 2015), and chloroplasts (Barton et al., 2018) present in this cortical domain exhibited a relatively slow and erratic movement with an average velocity of 0.75 μm s^–1^ for mitochondria and peroxisomes (Supplementary Figure 1). Chloroplasts in the epidermal cell cortex generally moved very slowly with occasional stops of up to several minutes before their position shifted again. Our earlier work has shown that such slow-moving chloroplasts are often located within pockets created by the subtending and closely appressed tonoplast (Barton et al., 2018).

Observations on the cortical regions of 375 un-stressed hypocotyl epidermal cells (5 cells/tissue × 25 seedlings) each from double transgenic (exhibiting combinations of chloroplast and mitochondria, chloroplasts and peroxisomes, and peroxisomes and mitochondria) and triple transgenic plants with fluorescently highlighted chloroplasts-mitochondria and peroxisomes showed that the three organelles were most often localized away from each other and exhibited independent movements. Time lapse imaging showed occasional cases where a mitochondrion or a peroxisome remained for 5–7 frames (10–14 s) around an epidermal chloroplast. However, observations of clear distances between the three different organelles did not suggest their sustained proximity within the cortical regions.

Nevertheless, a different impression was obtained when the three organelles were observed in cotyledon and leaf mesophyll cells of ten to twelve days old plants after an overnight period. In a top-down view both peroxisomes and mitochondria, ranging in diameters from 0.5 to 1.2 μm appeared packed between chloroplasts of 5 to 8 μm diameter. Notably, chloroplast movement was minimal in mesophyll cells taken after the dark period but resumed within 5 min of illumination with 120 ± 10 μmol m^–2^ s^–2^ light.

Based on observations on 50 cotyledon cells from 10 plants double transgenic for mitoGFP-Yperoxi, each cell showing between 12 to 16 chloroplasts in the top-down view, 1 to 3 of the small organelles were observed maintaining a dwell time higher than 20 s around a total of 196 chloroplasts (representing about 28% of the total number of chloroplasts observed). Within the same population only mitochondria were observed around 29 (ca. 13% of the total) of the chloroplasts, while 33 chloroplasts (ca. 17%) were surrounded only by peroxisomes and the rest exhibited both small organelles. Notably, as with the composition of the peri-plastid organelle cluster the relative positions of the small organelles continuously changed and conveyed the impression of their moving around a chloroplast.

Based on observations made on the cortical region of hypocotyl and cotyledon cells it was concluded that under normal conditions of growth, unstressed plants do not exhibit sustained proximity between chloroplasts, peroxisomes, and mitochondria. Whether this changed under stress was assessed next.

### Exposure to High Light Intensity Increases Clustering of Peroxisomes and Mitochondria Around Chloroplasts

Earlier our lab had reported increased peroxisome and mitochondrial interactions in response to high light stress (Jaipargas et al., 2016). Here we took 8–10 day old seedlings of a double transgenic (mitoGFP-Yperoxi) line after an overnight dark period and exposed them to high light intensity of 450 ± 25 μmol m^–2^s^–2^ for 5, 10 and 20 min before observations. Different from our earlier observations on plants growing under normal light conditions, exposing hypocotyl cells to 5 min to high light resulted in peroxisomes or mitochondria or both organelles clustering for more than 10 s around 36% of the larger mesophyll cell chloroplasts (MCC; *n* = 100) and 22% of pavement cell chloroplasts (PCC; *n* = 100). Increasing the exposure time to 10 min increased small organelle clustering to 78 and 50% around MCC and PCC, respectively (Figure 1B). A 20-min exposure increased clustering only slightly to about 55% around PCC (Figure 1B) but also resulted in 12–17% PCC displaying signs of chlorophyll bleaching (Figure 1C). Based on twelve separate instances of observing the three organelles moving together around the cell for up to 4 min, after 10 min of exposure it was concluded that high light induced stress increases proximity between the three organelles (Figure 1D).

Upon comparing the number of peroxisomes and mitochondria clustered around MCC and PCC in hypocotyl cells, it was found that more of the small organelles had appeared around MCC. While not experimentally assessed further, this observation might be due to the relatively large size (5–8 μm diameter) and high chlorophyll content of closely packed MCC, compared to the nearly-half sized (long axis approximately 2.5 μm), 9 to 12 PCC dispersed in a large epidermal cell (Barton et al., 2016).

**FIGURE 2:**
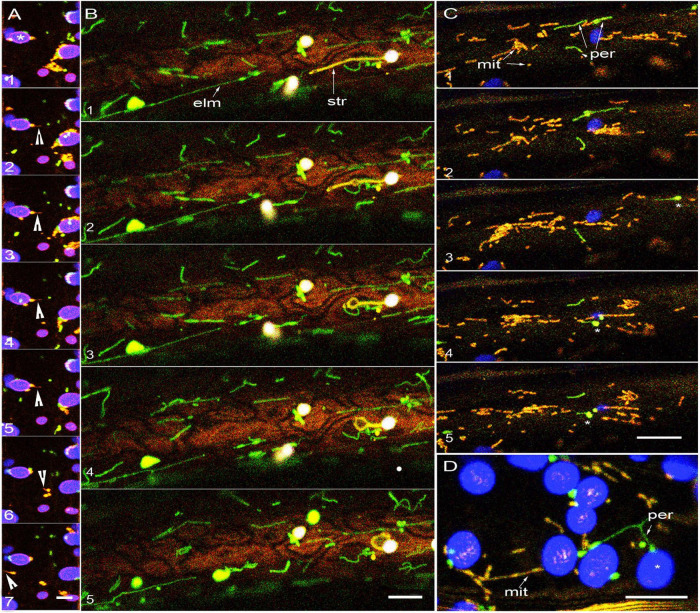
Tubular forms do not increase proximity between plastids, mitochondria and peroxisomes. **(A)** Sequential images taken after 5 min of exposure to about 450 μmol m^–2^s^–2^ of light show mild organelle clustering and a quick matrixule (arrowhead) extension response by a mitochondrion in apparent proximity to a chloroplast (*). Panel 7 and 8 show other mitochondria extending matrixules independently (arrowheads). While mitochondria and peroxisome remain around the lower chloroplast (*) the cluster around the other chloroplasts disperse over time suggesting that dwell time and organelle dynamics can vary considerably even in neighboring regions of the cell (see Supplementary Movies 2, 3). **(B)** Five sequential images from a single hypocotyl cell from a non-stressed double transgenic *elm1* mutant with elongated green fluorescent mitochondria (elm- arrow) and stroma targeted YFP. The elongated mitochondria do not interact with the extended stromule (str- arrow) that exhibits independent dynamic behavior (Supplementary Movie 4). **(C)** In non-stressed double transgenic *apm1* mutant elongated mitochondria (yellow -mit) and peroxisomes (per- green) pass by each other without showing sustained proximity. Peroxules (*) seen in panels 3, 4, 5 do not exhibit sustained proximity with the elongated mitochondria streaming around them (Supplementary Movie 5). **(D)** Representative snapshot from a double transgenic *apm1* mutant cell stressed by exposing to 450 ± 25 μmol m^–2^s^–2^ for 10 min suggests increased proximity between chloroplasts (* blue) elongated peroxisomes (per-green) and mitochondria (mit- yellow). Scale bars – A = 5; B, C, D = 10 microns.

Mesophyll cell chloroplasts, as the major organelles responsible for oxygenic photosynthesis in response to light are also active producers of reactive oxygen species (ROS; Foyer and Noctor, 2003; Noctor et al., 2018). The presence of peroxisomes around MCC in response to high light stress thus matches our earlier observations (Jaipargas et al., 2016). An increase in subcellular ROS results in the formation of peroxules, thin tubular extensions from peroxisomes (Sinclair et al., 2009). Similar tubular extensions called stromules and matrixules have been described from plastids and mitochondria, respectively (Scott et al., 2007; Mathur, 2021). Indeed, illuminating a triple transgenic SFR2mRFP-GPerox–Ymito line with 450 ± 25 μmol m^–2^s^–2^ for 5 min is sufficient to induce organelle clustering and the extension of matrixules (Figure 2A and Supplementary Movie 2). It has been suggested that since organelle extensions transiently change the shape of a spheroidal organelle into a more tubular form (Mathur, 2020) they may serve to increase interactivity between organelles (Mathur, 2021). While most of the observations on organelle extensions have used some form of stress to achieve their increased frequency (Mathur, 2021) a low number of tubular organelle forms is found under relatively non-stressed and normal growth conditions too (Barton et al., 2018). In light of our observations that the spheroidal forms of chloroplasts, peroxisomes, and mitochondria do not exhibit sustained proximity under non-stressed conditions we wondered if the presence of a tubular organelle form could increase its interactivity even under normal growth conditions. This idea was explored next using *Arabidopsis thaliana* plants with constitutively elongated organelles.

### Tubular Forms Do Not Increase Proximity Between Plastids, Mitochondria, and Peroxisomes

After an overnight period, chloroplasts in hypocotyl epidermal cells form long extensions within 2 h of illumination. A stable double transgenic line (mtyk-GPeroxi) that shows YFP-targeted to mitochondria and GFP-targeted to peroxisomes was crossed with another transgenic line expressing SFR2mRFP that labels the plastid outer envelope membrane (OEM) (Figure 2A and Supplementary Movie 3). The triple transgenic SFR2mRFP-GPerox–Ymito line showed long red fluorescent plastid extensions, green peroxisomes, and yellow mitochondria. Based on observations from 25 chloroplasts with extensions, we found that the smaller organelles formed transient clusters around the chlorophyll-containing plastid body with the longest dwell time of 270 s exhibited by a mitochondrion. By contrast, the longest dwell time for mitochondria and peroxisomes moving past plastid extensions of up to 30 μm was between 12–20 s. We concluded that the two small organelles did not exhibit prolonged proximity with plastid extensions. Whether two tubules would have increased proximity was investigated next.

The Arabidopsis *elongated mitochondria1 (elm1/nmt1)* mutant is impaired in mitochondrial fission and shows greatly elongated GFP-highlighted mitochondria (Arimura et al., 2008; Logan, 2010). The mutant was crossed with the PtYK line that carries a YFP targeted to the plastid stroma (Nelson et al., 2007). As depicted in Figure 2B and time lapse Supplementary Movie 4, observations on this double transgenic line established that 5 to 25 μm long yellow fluorescent plastid extensions were present within a few micrometers of the 5 to 50 μm long green fluorescent tubular mitochondria. As these elongated forms did not exhibit any intertwining or alignment we concluded that organelle tubulation does not necessarily increase organelle proximity.

A third probe was considered to observe the behavior of elongated peroxisomes and elongated mitochondria simultaneously. The Arabidopsis *apm1/drp3a* mutant is impaired in the fission of both peroxisomes and mitochondria and consequently both organelles appear abnormally elongated (Arimura et al., 2004; Mano et al., 2004). A double transgenic line called *apm1*-mtYK showed green fluorescent peroxisomes (Mano et al., 2004) and yellow fluorescent mitochondria (Figure 2). As demonstrated earlier (Jaipargas et al., 2016) peroxules interact with small, punctate mitochondria in response to high light induced stress. However, we did not observe tubular peroxisomes interacting with tubular mitochondria in the hypocotyl cells of 80 non-stressed *apm1*-mtyk plants ranging in age from 8 to 10 days. Although 12 independent instances were recorded in the cell cortex where both tubular forms appeared within a few μm of each other, they maintained independent dynamic behavior in a manner similar to their considerably smaller, non-tubular forms. Independent tubule behavior was further underscored by the non-synchronous extension and retraction of the tubules and the sporadic appearance of dilations along the tubule length (Figure 2C and Supplementary Movie 5).

Exposing the *apm1*-mtYK plants to 450 ± 25 μmol m^–2^s^–2^ for 10 min resulted in peroxisome and mitochondrial clustering around chloroplasts (Figure 2D) and occasional intertwining of the tubules. However, as demonstrated by us earlier (Schattat et al., 2012) the intertwining and close interaction of tubular mitochondria results in their fusion and exchange of matrix proteins. Here, instead of suggesting increased proximity leading to interactions, the dynamic folds and loops formed asynchronously by the tubular organelles only served to reinforce their independent behavior.

A triple transgenic line expressing yellow fluorescent mitochondria (Ymito/mt-Yk; Nelson et al., 2007), and *SFR2*mRFP that highlights the plastid OEM in red was created in the *apm1* mutant background that already possesses elongated green fluorescent peroxisomes (Mano et al., 2004). Although all three organelles showed tubules, and four instances of elongated peroxisomes and mitochondria moving in tandem within a cytoplasmic strand were noted, two or three tubules representing the different organelles remaining in close proximity were not observed. As noted earlier in the *apm1*-mtYk line, hypoxia resulted in mitochondrial expansion into assorted shapes but there was only a decrease in the extension-retraction of plastid extensions and the dynamic movement of tubular peroxisomes.

We concluded that under normal conditions of growth both the spherical-oblong as well as the tubular forms of chloroplasts, mitochondria and peroxisomes maintain their spatiotemporal independence. Increased proximity that could suggest their high interactivity was not observed. While high light intensity-induced stress reduced the distance between the three organelles, hypoxia did not change their separate localization but largely affected their dynamic behavior.

The conundrum facing us at this stage was that our observations on the three key organelles in living cells contradicted the proximity-based suggestions used to underscore their biochemical interactivity. Although their increased interactivity was observed in response to photorespiratory stress, it seemed implausible to consider that chloroplasts, mitochondria and peroxisomes do not come close to each other under non-stressed conditions. An additional question was about the mechanism by which their spatial relationship changed quickly in response to stress. Therefore, we considered the possibility that instead of relying upon direct contacts with each other the interactions between these organelles involved a common mediator.

### Irrespective of Their Shapes Plastids, Mitochondria, and Peroxisomes Are Closely Appressed to the Endoplasmic Reticulum Membranes

**FIGURE 3:**
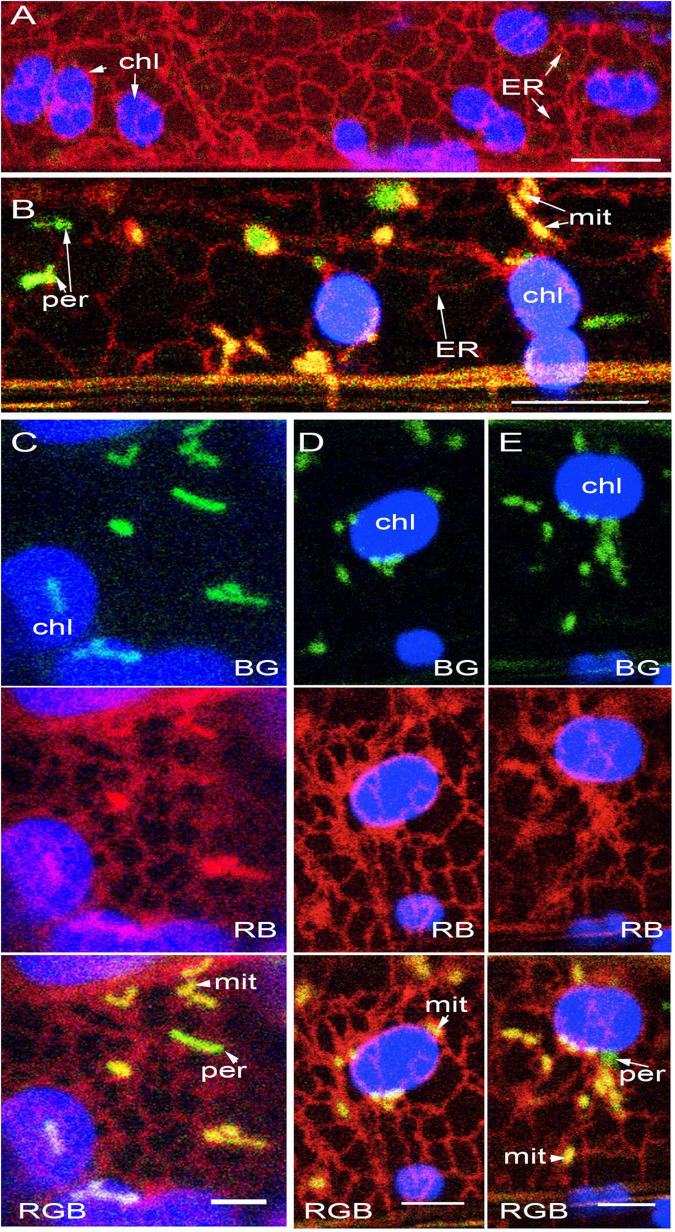
Plastids, mitochondria and peroxisomes are individually surrounded by the ER. **(A)** Representative image showing the general ER mesh(red) and the loose ER-cage formed around chloroplasts (chl). **(B)** A representative image from GPeroxi-YMito-RER plants showing chloroplasts (chl in blue), mitochondria (mit in yellow) and peroxisomes (per in green) individually enmeshed in the ER (red). **(C)** Representative snapshot showing the independent spatial relationship that the ER (red) maintains with chloroplasts (chl-blue), mitochondria (mit- yellow) and peroxisomes (per -green). **(D)** Mitochondrial clusters in the peri-chloroplastidic ER and neighboring regions with each mitochondrion encased in its own ER. **(E)** Mitochondrial (mito – yellow) and peroxisomal (per- green) clusters in the peri-chloroplastidic ER (red)and neighboring regions with each of the smaller organelles encased in its own ER pocket (Based on Supplementary Movie 6). Scale bars- A, B = 10; C, D, E = 5 μm.

We have reported earlier on the independent alignments between the endoplasmic reticulum (ER) and peroxisomes (Sinclair et al., 2009; Barton et al., 2013, 2014), the ER and plastids (Schattat et al., 2011a,b; Barton et al., 2018) and the ER and mitochondria (Jaipargas et al., 2015, 2016). Observations made using different double transgenic lines, GPeroxi-RER that showed peroxisomes in green and the ER tubules in red, (mitoGFP)-RER that showed green mitochondria and red fluorescent ER lumen and (tpFNRG-EGFP)-RER where plastid extensions appeared green and the ER lumen appeared red, reconfirmed that each of the three key organelles is independently enmeshed in the ER (Figures 3A–E). While the mesh around mitochondria and peroxisomes is observed in Figures 3B,C shows peroxisomes and mitochondria at a distance from the chloroplast. A cluster of mitochondria (Figure 3D) and of mitochondria and peroxisomes (Figure 3E) is observed around separate chloroplasts. While showing the ER sandwiched between the different organelles, the observations also established that each cluster of small organelles around chloroplasts could be different from the other.

**FIGURE 4:**
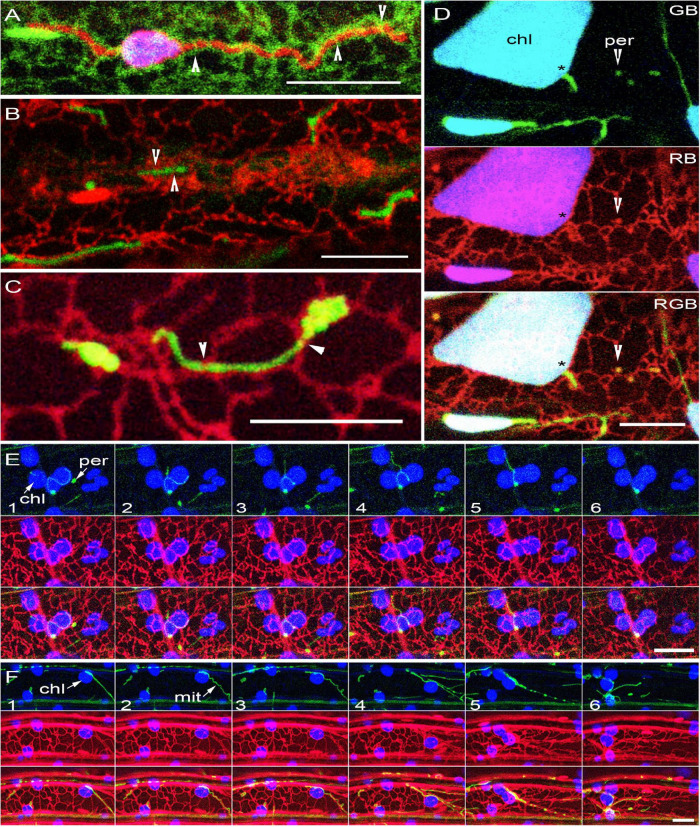
Organelle extensions and tubular forms maintain connectivity only with the ER. **(A)** Plastid extension highlighted by OEM targeted expression of SFR2mRFP (red) enmeshed in the ER (green). Note the ER bands encircling the tubular extension (arrowheads). **(B)** Elongated mitochondria (green) in the Arabidopsis *elm1* mutant enmeshed in the ER (red) through the ER encircling the tubules at different points (arrowheads). **(C)** GFP-labeled peroxisomes in the Arabidopsis *apm1* mutant enmeshed in the ER (red) with main contact points indicated by arrowheads. **(D)** A single snapshot from image series shows large chloroplasts in the Arabidopsis *arc6* mutant with extensions (*), highlighted using a stroma targeted tpFNR-EGFP and observed with peroxisomes (yellow -arrowheads), both enmeshed independently by the ER (red). Chlorophyll depicted in blue (B), GFP in green (G), RFP in red (R); Yellow color of peroxisomes detected by overlapping R and G fluorescence. **(E)** Six sequential images showing an elongated green fluorescent peroxisome (per) in the Arabidopsis *apm1* mutant along with red fluorescent ER and chloroplasts (chl- blue). Note the close association between the peroxisome and the peri-plastid ER which allows the peroxisome to appear close to and molded around the chloroplasts. **(F)** Six sequential images from a time lapse series (Supplementary Movie 7) of elongated mitochondria (mit) in Arabidopsis *elm1* mutant appearing closely appressed to the chloroplast (chl- blue) as both organelles relocate in tandem with changing ER (red) organization. Scale bars = 10 μm.

The ER mesh around chloroplast extensions (Figure 4A), tubular mitochondria (Figure 4B), and tubular peroxisomes (Figure 4C) reinforced the idea that the ER formed the most extensive membrane network around the different organelles. Additional observations came from a triple transgenic line in the Arabidopsis *accumulation and replication of chloroplasts 6* (*arc6*; Pyke et al., 1994) mutant background. Compared to chloroplasts in wild type Arabidopsis plants, the 2–3 mesophyll chloroplasts in the *arc6* mutant may show an enlarged size of up to 25 times and have a higher frequency of long plastid extensions (Pyke et al., 1994; Holzinger et al., 2008). The triple transgenic *arc6* tpFNREGFP-RER – Yperoxi line allowed simultaneous assessment of the spatial relationship between individual peroxisomes and the long plastid extensions with the surrounding ER (Figure 4D). Time lapse movies established that ER-enmeshed peroxisomes could occasionally align over a few μm with the tubular extensions or move past the extensions following a different trajectory. However, even when they appeared near each other, both the plastid extensions and the peroxisomes remained encaged independently by the ER.

Further evidence on the close alignment between the ER and tubular forms came from several other transgenic lines: the *apm1*-RER that showed elongated peroxisomes in green and the ER tubules in red (Figure 4E), *elm1*-RER that exhibited elongated green mitochondria and ER in red (Figure 4F). Since the *apm1* and the *elm1* mutants have elongated green fluorescent peroxisomes and mitochondria, respectively, time lapse image sequences showed how ER rearrangement affected the enmeshed organelles and their relation with the chloroplasts (Figures 4E,F). For both organelles, observations based on GFP and chlorophyll autofluorescence (e.g., top panels in Figures 4E,F) only showed the close alignment of the tubular organelles around the chloroplasts. Visualization of the ER created a different impression as it filled in the ER membranes present between the organelles.

We concluded that the ER is sandwiched between and in contact with each of the organelles considered here. The intimate association with the ER suggested that a change in ER-dynamics could directly impact the spatiotemporal behavior of the organelles embedded in it.

### Conditions That Alter ER Membranes Also Affect Plastids, Peroxisomes, and Mitochondria

As shown earlier (Figure 1) exposure to high light intensity had increased the proximity between chloroplasts, mitochondria, and peroxisomes, although each organelle was in a separate ER pocket (Figures 3, 4). Observations on 28 cells in 7 plants of the Gmito-Yperoxi-RER triple transgenic line showed that exposure to high light intensity for 10 min significantly altered the size of ER polygons around the different organelles. In an earlier publication we have reported that ER polygon sizes change in response to dark and light conditions and correlate with the fission of mitochondria (Jaipargas et al., 2015). Here, we observed that peroxisomes and mitochondria became confined within small ER polygons that developed around chloroplasts (Figures 3D,E). While this phenomenon requires further detailed investigations at the ultrastructural level, it suggested that closer contacts could occur between the membranes of the ER and the enmeshed organelles.

**FIGURE 5:**
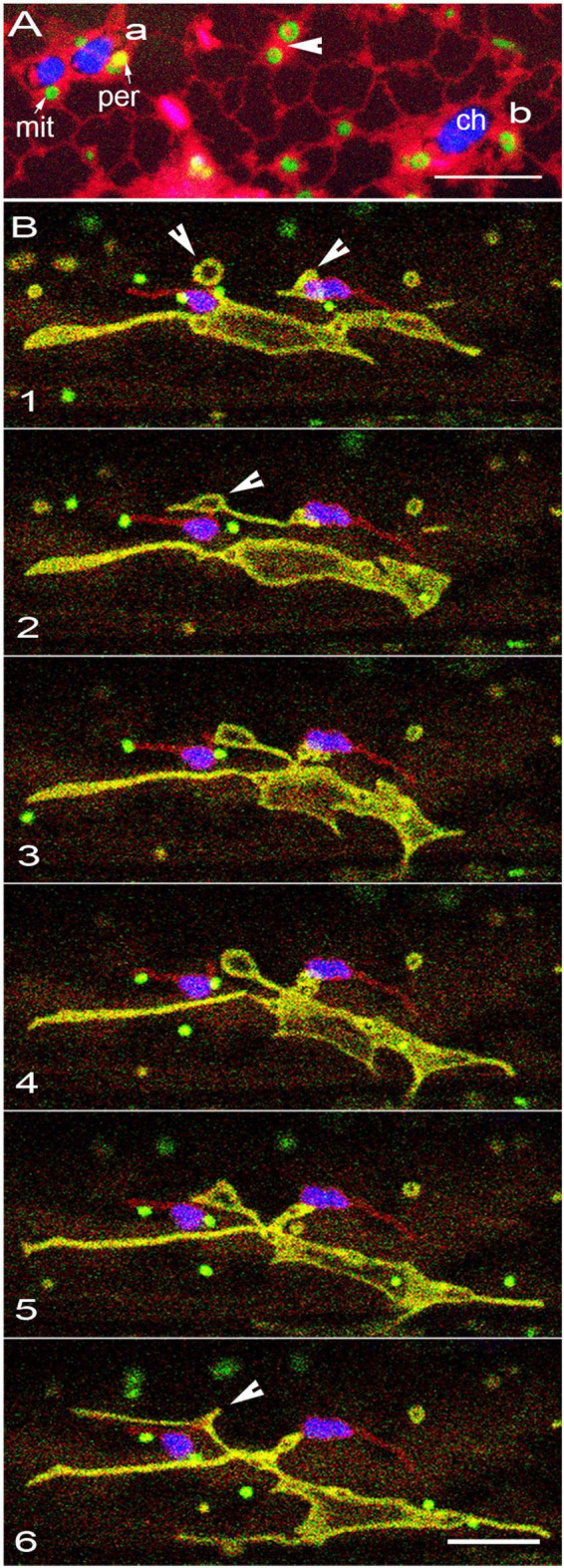
Changes in ER membranes coincide with alterations in the morphology and behavior of other organelles. **(A)** Representative image of organelles in GPeroxi-YMito-RER triple transgenic plants under hypoxia after 45 min under water. While the ER-tubules (red) have not disappeared completely, chloroplasts (blue – chlorophyll; ch), peroxisomes (yellow – per) and mitochondria (mit – green) become surrounded separately by expanded, thin ER membranes (red). Whereas some regions of the expanded ER may not exhibit entrapped organelles, in other locations such as, “a” three organelles are trapped, “b” shows only a chloroplast and a mitochondrion while an arrowhead indicates two swollen, donut -shaped mitochondria (arrowheads). The phenomenon is reversible. **(B)** Six sequential snapshots from a time-lapse series (Supplementary Movie 8) illustrate the pleomorphy of expanded dynamic mitochondria while peroxisomes (green) and plastids (red OEM) retain their shapes and motility. While some donut-shaped mitochondria remain independent, other mitochondria indicated by arrowheads (panels 1, 2) fuse over time (arrowhead panel 6) to form a single large mitochondrion. Scale bars = 10 μm.

An opposite effect on the ER membranes was observed in seedlings placed in water under a glass coverslip for 45 min. This treatment creates hypoxic conditions for the plant cells (Van Gestel and Verbelen, 2002; Jaipargas et al., 2015). As observed here, the movement of organelles showed a general slowing down from ca. 1.5 μm s^–1^ to between 0.75–0.5 μm s^–1^. The most pronounced effect was observed on mitochondria which, during their slow movement, also expanded and fused to form giant mitochondria (Figures 5A,B). Concomitantly, the ER membranes expanded and thin cisternae became more prominent than the tubules. We concluded that ER membrane expansion was the reason for the reduced organelle motility. Notably the slowed down motility of all organelles allowed their independent behaviors to be maintained (Figure 5B) and did not increase their proximity.

In plant cells, it is well established that the cytoskeleton provides the tracks for movement of all organelles including the endomembrane system (Boevink et al., 1998; Nebenführ et al., 1999; Mathur et al., 2002). While an impaired actin cytoskeleton results in a general collapse of organelle movement (Mathur et al., 2002, 2003) we wished to assess whether peroxisomes and mitochondria came closer to chloroplasts during this process. The effects of Latrunculin B, a well-established inhibitor of actin polymerization (Spector et al., 1989) on organelle proximity were investigated. Treatment of the triple transgenic line (Gmito-Yperoxi-RER), with 100 nM of Latrunculin B for an hour resulted in a disruption of ER tubules, the formation of large diffuse globules, the development of a diffuse red fluorescence in the cell, and a general cessation of organelle motility. The effects of a general collapse of actin organization and the clumping of organelles were similar to earlier reported observations (Mathur et al., 2002; Barton et al., 2013) and have therefore not been shown here. Notably, mitochondrial expansion that formed a prominent feature of the response to hypoxia was not observed. It was concluded that while the other two treatments directly seemed to affect ER membranes the change in organelle proximity due to impaired actin cytoskeleton was a non-specific effect reflecting a totally collapsed subcellular activity.

Taken together, our observations reaffirmed that organelle movement, including the dynamic organization of the ER are actin dependent processes. Nevertheless, compared to the phenomenon of organelle motility the decrease in ER polygon size under high light as well as the expansion of ER membranes under hypoxia might be more directly linked to rapid alterations in organelle membranes. While these observations require more detailed investigations, it was concluded here that changes that result in the swelling or fusion of ER membrane have a direct effect on the distance between enmeshed organelles.

## Discussion

Our investigations started as an innocuous attempt to understand the routinely used words, ‘organelle interactivity.’ The words often create a useful backdrop for explaining the numerous biochemical interactions and pathways necessary for cell survival and proliferation. For many biologists, the words also evoke an image of the interacting organelles being close to each other. Despite the widely held belief in organelle interactivity being linked to proximity, the actual evidence behind the idea was traceable to only a few high-resolution ultrastructural snapshots of plant cells [e.g., Frederick and Newcomb (1969); Frederick et al. (1975); Hayashi et al., 2001; Sage and Sage, 2009]. Observations from studies using fluorescent fusion proteins to look at living plant cells have also contributed to the belief. Many of the studies use transient protein over-expression systems over incredibly long durations and frequently employ excised, wounded tissue for drawing conclusions about protein localizations and organelle interactions. Nevertheless, even with their accompanying artifacts, observations on stressed plant cells have been relevant in building up our ideas about subcellular interactions in the plant cell. One of the insights from observing living plant cells is that the cell continues to respond to the stimulus and consequently organelle behavior changes very quickly.

In order to add more insights to the body of knowledge on organelle interactions this study took a purely observational approach to find out about the actual proximity between different key organelles in living, intact plant cells under normal conditions of growth and development.

We considered that our baseline observations would lay down the foundation for more detailed studies leading to the discovery of proteins involved in creating MCS and exchange channels between organelles. Unexpectedly, our observations on non-stressed cells did not show close associations between chloroplasts, mitochondria, and peroxisomes, the three key organelles implicated in myriad aspects of plant metabolism. Whereas even transient stress can bring some of these organelles closer to each other and maintain proximity for several minutes it became clear that the majority of organelles exhibit a rather individualistic behavior. What allows a few chloroplasts, mitochondria, and peroxisomes to come together while others remain at a distance from each other?

We found that each of the three organelles investigated by us, irrespective of the form that they transiently adopted, did not interact directly with each other. Instead, each of them remained in close connectivity with the ER. Notably, exposure to high light intensity affects all the organelles and results in an increase in subcellular ROS. A short random diffusion distance of 1 μm is attributed to H_2_O_2_ and other ROS (Hallwell and Gutteridge, 1989) and one of the responses to increased cellular ROS is the expansion of membranes (Margittai et al., 2008). Based on our observations in response to high light intensity and hypoxia we suggest that changes in ER membrane dynamics are responsible for changes in the proximity of enmeshed organelles. Most significantly, even when the organelles appear close to each other and form clusters that move around the cell, the ER membranes were always present between them.

This study only observed the relationship between the ER and chloroplasts, mitochondria, and peroxisomes. Within the limitations imposed by light microscopy, but with confidence due to the clear distance maintained between the majority of organelles observed, we surmised that the three key organelles do not interact physically with each other. Whether the ER is also involved in creating peri-nuclear clusters of chloroplasts (Erickson et al., 2017; Ding et al., 2019) remains an open question at present. For a similar reason, a more detailed investigation is underway for exploring the intimate relationship between peroxisomes and lipid droplets (LD; Thazar-Poulot et al., 2015; Cui et al., 2016). It is possible that similar to the relationship between the ER and Golgi bodies (Brandizzi et al., 2002; Sparkes et al., 2009; Stefano et al., 2012) peroxisomes and LD and other *de novo* generated endomembrane derived organelles and vesicles do exhibit direct interactivity with each other.

While our findings do not challenge the biochemical interactivity known to exist between different organelles, they do introduce some additional membrane layers that need to be accounted for during the exchanges.

Perhaps a key to assimilating our new insights into existing knowledge would lie in understanding the general redox state of a cell but then also breaking it down into multiple localized events so that the formation of small, transient redox domains may also be considered.

As already well established for mitochondria (Rizzuto et al., 1998; Kornmann et al., 2009; Csordás et al., 2010) MCS between the ER and the different enmeshed organelles might be the norm. Consequently, the specific output from an organelle (for example different forms of ROS; Exposito-Rodriguez et al., 2017) in response to a stimulus would affect the contiguous ER to produce an ER-microdomain. The continuous reorganization of ER tubules and cisternae would facilitate interactions between such *de novo* generated ER-microdomains. Thus, we suggest that instead of direct physical interactions occurring between the membranes of functionally discrete organelles, it is the membranes of ER-microdomains that interact with each other. The cumulative response output of a plant cell would still reflect the activities of all the different organelles but its dispersal and dissemination would involve interactions of ER-microdomains.

## Conclusion

Although a compelling number of TEM images in published literature suggest direct interactions between different organelles our imaging of living plant cells using a variety of mutants and double and triple transgenic lines expressing targeted fluorescent probes did not convince us of their direct connectivity. Under non-stressed conditions a majority of organelles did not even come near each other. However, our observations clearly established that irrespective of their form at any given time, all plastids, mitochondria and peroxisomes remained in intimate connectivity with ER membranes. Whether ER mediation is a global phenomenon applicable to the interactivity of all organelles in a cell remains to be established. The use of mutants such as *ermo1/gnl1* (Nakano et al., 2009), *gom8* and *rhd3-7* (Stefano et al., 2012) that are impaired in ER morphology and dynamics should provide interesting insights on the role played by the ER in dictating the behavior and interactions of other organelles. Since many more insights might be obtained by using the robust set of new cell biological tools generated during this study we invite other investigators to undertake more detailed investigations on subcellular interactions in the plant cell.

## Materials and Methods

### Gene Cloning

PCR primers for cloning the outer envelope localized gene SENSITIVE TO FREEZING2 (SFR2; Moellering et al., 2010) AT3G06510 gene were based on the coding sequence available in the Arabidopsis Information resource <TAIR; https://www.arabidopsis.org/> (Forward primer JM698 5′GTC TAGAATGGAATTATTCGCATTGTTAATT 3′carrying an *Xba*I site and the reverse primer with *Bam*HI site introduced – JM 699 5′GGATCCGTCAAAGGGTGAGGCTAAAGCA 3′. The resultant CDS was ligated in frame to create a C-terminal fusion with a monomeric RFP (mRFP; Campbell et al., 2002) and placed in a binary pCAMBIA 1300 vector backbone to which a proCaMV-35S and a nos-terminator sequence had been added earlier.

### Creation of Transgenic Plants and Growth Conditions

Single and double transgenic lines of *Arabidopsis thaliana* were created through *Agrobacterium tumefaciens* mediated floral dip transformation (Clough and Bent, 1998) or through crossing of already selected stable transgenic lines. The proCaMV35S-SFR2mRFP transgenic lines were created in the Columbia ecotype of *Arabidopsis thaliana*. Other lines used have been published earlier and used for double or triple transgenic plants as detailed in Table 1. All seeds were stratified for 2 days at 4°C. For investigating soil-grown plants Sunshine mix LA4 (Sun Gro Horticulture, United States) soil in sealed Magenta boxes was used. Alternatively, if specified, plants were grown in plastic petri dishes containing Murashige and Skoog basal medium (Murashige and Skoog, 1962; Sigma M404) with B5 vitamins, 0.75% sucrose and 3 g/L phytagel (Sigma-Aldrich) (pH adjusted to 5.8 before autoclaving). Plants were grown for 7–14 days under a long-day light regime (16-h-light–8-h- dark) under light intensity of 120 ± 20 μmol m^–2^ s^–1^ light and an ambient temperature of 21 ± 2°C.

### Microscopy

Entire seedlings were lifted from the soil, mounted in deionized water (pH 6.9 ± 0.2) on a glass depression slide and placed under a coverslip for observation using a 40X (numerical aperture 0.80) water immersion ceramic lens. A three channel Leica TCS-SP5 confocal laser- scanning unit equipped with 488 nm Ar and 543 nm He-Ne lasers was used for simultaneous imaging of GFP, YFP, RFP, and chlorophyll. The emission spectra acquired were: GFP—503 to 515 nm (green); RFP- 555 to 630 nm (red); chlorophyll—650 to 710 nm (false colored blue). In double and triple transgenic plants with a YFP probe the yellow fluorescence was detected by extending the Red channel acquisition (525 to 630 nm) to create an overlap with GFP emission spectrum. The merged regions in images appeared yellow and were confirmed separately by using a YFP-dedicated 514 nm excitation laser line.

The x,y,z series used for 3-D projections maintained a 0.99 μm distance between sections. Time-lapse image capture (x, y, t) used a 1024 × 512-pixel box size and a line averaging of three in the bi-directional mode to obtain a frame every 1.935 s. All images were captured at a color depth of 24-bit RGB, cropped and processed for brightness/contrast as complete montages or image stacks using either Adobe Photoshop CS3^1^ or the ImageJ/Fiji platform^2^. Adobe Photoshop was used for annotation of images and movies.

### Measurement of Lipid Droplets Dwell Time Around Chloroplasts

In our experiments the idea of measuring “dwell time” was to establish the time that two organelles stayed together within a close distance. For measuring the dwell time at least ten independent time-lapse series ranging from 120 to 240 s (about 60 to 120 frames of 1024 × 512 pixels each) were used. Recognizing that in living cells the relative velocity of organelles is not constant (Mathur et al., 2002), it was assumed that a 1 μm diameter organelle, moving at a low velocity of 0.5 μm s^–1^ would require 20 s to move past a relatively immotile 8 μm diameter organelle and a buffer zone of 1 μm on each side (total path length of 10 μm). The Leica TCS SP5 point scanning laser confocal system used by us requires nearly 20 s for 10 x, y, t scans of a 1024 × 512 pixels rectangle with each scan an averaging of 3 lines. A “dwell time” longer than expected by the relative dimensions of the organelles was considered as being beyond a coincidental occurrence where the two organelles just happened to be in the same cytoplasmic neighborhood at the same time. While dwell time does not inform about whether an interaction has taken place or not it provides a likelihood for interaction with the chances of organelle interactions increasing with longer dwell time.

### Stress and Inhibitor Treatments

High light stress was created on the microscope stage using the incandescent lap used routinely for bright field imaging an intensity of 450 ± 25 μmol m^–2^ s^–2^ for varying amounts of time. Mild hypoxic conditions were routinely obtained within 45 min of seedlings being placed in water under a 24 × 60 mm glass cover slip (Fisher Sci. 12-548-5P) and were characterized by the appearance of swollen mitochondria. Actin polymerization inhibition was achieved by treating the different seedlings for 1 h with 100 nM Latrunculin B (CAS 76343-94-7- Calbiochem) and observing them while still in the inhibitor solution.

## Data Availability Statement

The raw data supporting the conclusions of this article will be made available by the authors, without undue reservation.

## Author Contributions

JM initiated the study and designed the experiments. OK helped with the imaging. ML cloned the SFR2 gene. NM did all the plant transformations and growth experiments. All authors contributed to the article and approved the submitted version.

## Conflict of Interest

The authors declare that the research was conducted in the absence of any commercial or financial relationships that could be construed as a potential conflict of interest.

## Publisher’s Note

All claims expressed in this article are solely those of the authors and do not necessarily represent those of their affiliated organizations, or those of the publisher, the editors and the reviewers. Any product that may be evaluated in this article, or claim that may be made by its manufacturer, is not guaranteed or endorsed by the publisher.

## Footnotes

1
http://www.adobe.com

2
http://fiji.sc/Fiji

## Supplementary Material

Supplementary Figure 1 Nine successive frames taken at intervals of 3 s from one of the 25 independent time-lapse x,y,t series taken on transgenic mtYK-Gperoxi plants that were used to observe differences in the rate of organelle movement in transvacuolar cytoplasmic strands (indicated by white arrow) compared to the cortical cytoplasmic sleeve. Small organelles often cluster in the linear cytoplasmic strands (arrowheads in different panels) and move at nearly twice the speed in comparison to the random and erratic movements of organelles in the cortical regions (peroxisome, red circle, mitochondrion white square). However, transvacuolar strands undergo rearrangements ly and small organelles are frequently drawn into the flow to move at a faster rate. Size bar = 10 μm.

Supplementary Movie 1 A representative time lapse (x,y,t) series comprising of 135 frames representing about 270 s of imaging time played at 3 fps. The series was captured from a single hypocotyl cell from a non-stressed triple transgenic Arabidopsis exhibiting the plastid OEM labeled by SFR2mRFP, peroxisomes targeted using GFP-SKL (Mathur et al., 2002) and mitochondria targeted with a YFP (Nelson et al., 2007). The chloroplasts, with chlorophyll false colored blue, exhibit OEM extensions. Numerous mitochondria and peroxisomes pass by the extensions and in a few frames appear to cluster around the chloroplast body. However, the subcellular motility presented in these images do not convey an impression of sustained proximity.

Supplementary Movie 2 A representative x,y,t series depicting 60 frames and a collective time of about 120 s shows mild organelle clustering after a 5 min exposure to about 450 μmol m^–2^ s^–2^ of light. The number of mitochondria and peroxisomes that appear to cluster around chloroplasts and elsewhere in the cytoplasm keeps on changing over time. The small organelles maintain proximity near a single chloroplast in the lower part of the images for the duration of the image series collection. The movie illustrates that the response of different organelles with respect to each other including the dwell time between them can vary organelles considerably within a small subcellular locale. It also demonstrates that matrixules may be extended by independent mitochondria and do not necessarily interact with chloroplasts or peroxisomes in the neighborhood.

Supplementary Movie 3 A time lapse series acquired over 140 s on a hypocotyl cell with plastid extensions (red), mitochondria and peroxisomes shows that the smaller organelles have a higher likelihood of interacting with the plastid body than with the long tubular extensions. Sustained proximity between mitochondria (yellow) and peroxisomes (green) is not concluded.

Supplementary Movie 4 A 1:05 min x,y,t series of elongated peroxisomes (green) and mitochondria (yellow) in a hypocotyl cell of double transgenic *apm1* mutant establishes that the tubulation of both organelles does not prolong their proximity with each other. As seen for their non-elongated forms both organelles maintain independent behavior, including the sporadic formation of loops, constrictions and dilations along tubule length.

Supplementary Movie 5 A 76 s time lapse representing 228 s of acquisition time from a double transgenic line in the *elm1* mutants shows green mitochondria and yellow plastid extensions. Despite both organelles presenting tubules within a few micrometers of each other, the tubules behave independently and do not exhibit increased proximity with each other. The plastid extension changes its shape considerably during the image acquisition time but this does not have an influence on its interactivity with elongated mitochondria.

Supplementary Movie 6 A x,y,t series of 39 s representing about 118 frames shows a single cell from a triple transgenic plant expressing ER in red, mitochondria in green and mitochondria in yellow. Chlorophyl autofluorescence has been depicted in blue. Peroxisomes and mitochondria appear around the chloroplast but occupy separate pockets of the ER present within the peri-plastidic ER cage. Thus, their proximity to the chloroplasts results in ER being sandwiched between the three different organelles.

Supplementary Movie 7 Time lapse series capturing about 387 s of elongated mitochondria (green) and ER (red) behavior in relation to chloroplasts (depicted blue) in a hypocotyl cell of the *elm1* mutant. The long mitochondria appear to wind around a chloroplast and faithfully move during its translocation. However, the time lapse sequence reveals that it is the intimate association of both chloroplasts and mitochondria with the ER that is responsible for their proximity and coordinated movement.

Supplementary Movie 8 A time-lapse series captured over 225 s shows the differing effects of hypoxia on mitochondria (yellow) that expand considerably into assorted shapes that fuse and extend or retract. Plastid extensions (red) do not change while all organelles including peroxisomes (green) continue moving independently.
