# Supplementary figures and images for: The ER Is a Common Mediator for the Behavior and Interactions of Other Organelles
Source: Front Plant Sci. 2022 Mar 25;13:846970. doi: 10.3389/fpls.2022.846970 (PMC8990311; doi:10.3389/fpls.2022.846970)

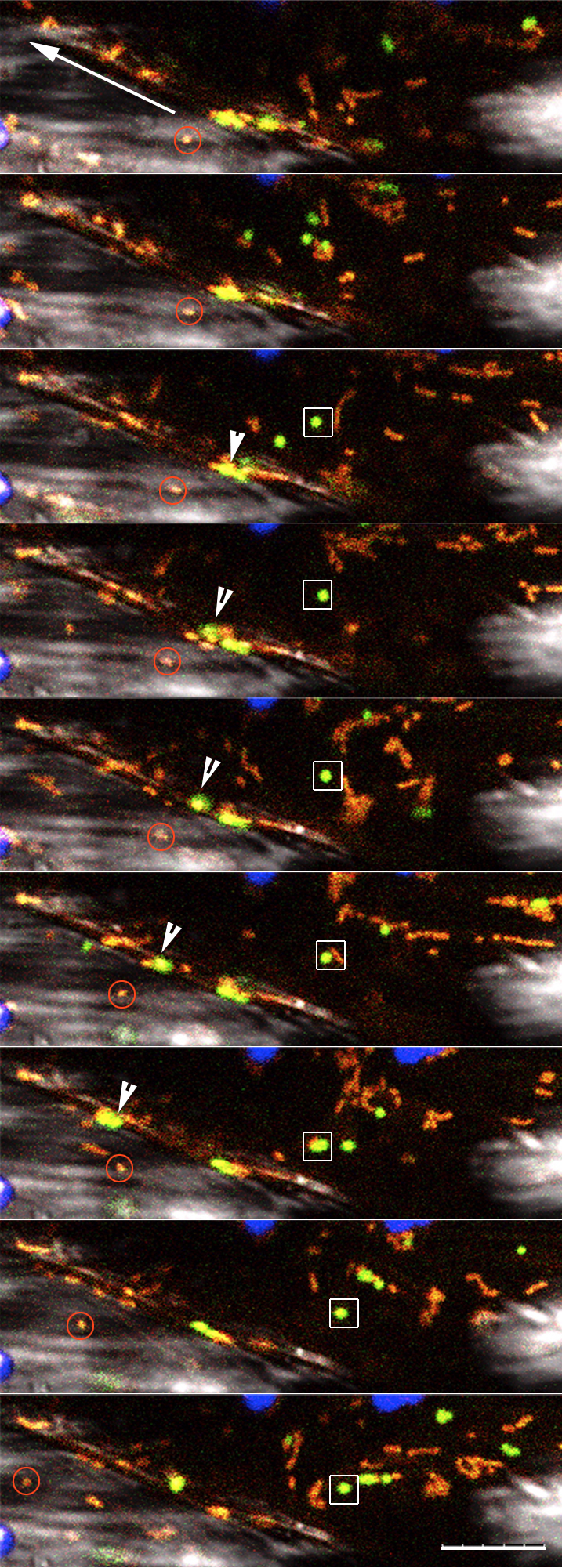

Supplement: Supplementary Figure 1 — Nine successive frames taken at intervals of 3 s from one of the 25 independent time-lapse x,y,t series taken on transgenic mtYK-Gperoxi plants that were used to observe differences in the rate of organelle movement in transvacuolar cytoplasmic strands (indicated by white arrow) compared to the cortical cytoplasmic sleeve. Small organelles often cluster in the linear cytoplasmic strands (arrowheads in different panels) and move at nearly twice the speed in comparison to the random and erratic movements of organelles in the cortical regions (peroxisome, red circle, mitochondrion white square). However, transvacuolar strands undergo rearrangements ly and small organelles are frequently drawn into the flow to move at a faster rate. Size bar = 10 μm. [file Image_1.JPEG]
